# Identification of a novel *CRYAB* mutation associated with autosomal recessive juvenile cataract in a Saudi family

**Published:** 2009-05-15

**Authors:** L Abu Safieh, AO Khan, FS Alkuraya

**Affiliations:** 1 Department of Genetics, King Faisal Specialist Hospital and Research Center, Riyadh, Saudi Arabia; 2 Department of Pediatric Ophthalmology, King Khaled Eye Specialist Hospital, Riyadh, Saudi Arabia; 3 Department of Pediatrics, King Khalid University Hospital and College of Medicine, King Saud University, Riyadh, Saudi Arabia; 4 Department of Anatomy and Cell Biology, College of Medicine, Alfaisal University, Riyadh, Saudi Arabia

## Abstract

**Purpose:**

To describe the first cataract-causing recessive mutation in the crystalline, alpha-b gene *CRYAB.*

**Methods:**

Homozygosity mapping complemented by linkage analysis was performed in a family with autosomal recessive juvenile cataract.

**Results:**

A homozygous missense mutation in *CRYAB* was identified. The mutation replaces a highly conserved amino acid residue in a dual function domain of the protein. None of the patients has clinically significant myopathy, but the oldest patient (the mother) has retinal pathology.

**Conclusions:**

This is the first report of a recessive mutation in *CRYAB* causing cataract.  Based on recent knowledge of the structure and function of this small heat shock protein, we speculate on the potential mutational mechanism.

## Introduction

Cataract is a clinically and genetically heterogeneous eye disease in which the lens loses its transparency [1]. Congenital and juvenile cataracts, much less frequent than the senile form, are especially damaging to the visual field because the visual cortex will adapt irreversibly to long-term lack of visual input from the affected eye, a condition referred to as amblyopia [2]. While infections represent an important cause of congenital and juvenile cataracts (particularly in underdeveloped countries), a genetic etiology can be identified in up to 50% of the cases. Although all forms of Mendelian inheritance have been reported, autosomal dominant inheritance is the most common [3]. An exception is observed in highly consanguineous populations where autosomal recessive forms are relatively more common and may even dominate the genetic landscape of the hereditary forms of congenital and juvenile cataract [4].

Crystallins are by far the most abundant lens soluble proteins, and their structure and organization are perfectly suited to their function of maintaining lens transparency [5]. It is not surprising, therefore, that mutations in this family of proteins can cause cataract. In fact, almost half of hereditary cataracts can be traced to mutations in one of the several crystallin genes [2]. Of three classes of crystallins found in vertebrate lenses, α-crystallin is the most abundant. It forms a unique cytoskeletal filament that is a stable complex of both αB- and αA-crystallin as well as lens intermediate filaments [6]. αB-crystallin, encoded by *CRYAB*, has an unusually broad tissue distribution profile among the crystallins that are highly enriched in the lens. The expression of *CRYAB* has been observed in the retina as well as in the cardiac, kidney, and skeletal muscles where it likely performs a cellular function beyond the optical transparency it provides in the lens [7]. Not surprisingly, mutations in *CRYAB* can cause cataract as part of a desmin-related myopathy phenotype [8,9]. All previously reported *CRYAB* mutations are dominant, but the exact mutational mechanism is not known.

In this study, we report linkage data on a family with autosomal recessive juvenile cataract. Not only do we show the underlying genetic defect to be the first reported autosomal recessive mutation in *CRYAB*, but we also document retinal involvement in the oldest affected individual (the mother). This novel mutation has no apparent clinical effect on cardiac or skeletal muscle function and displays variable expressivity. We discuss how this mutation broadens the phenotype associated with *CRYAB* mutations and how it may improve our understanding of the mutational mechanism of previously reported dominant mutations.

## Methods

### Human subjects

**Figure 1:**
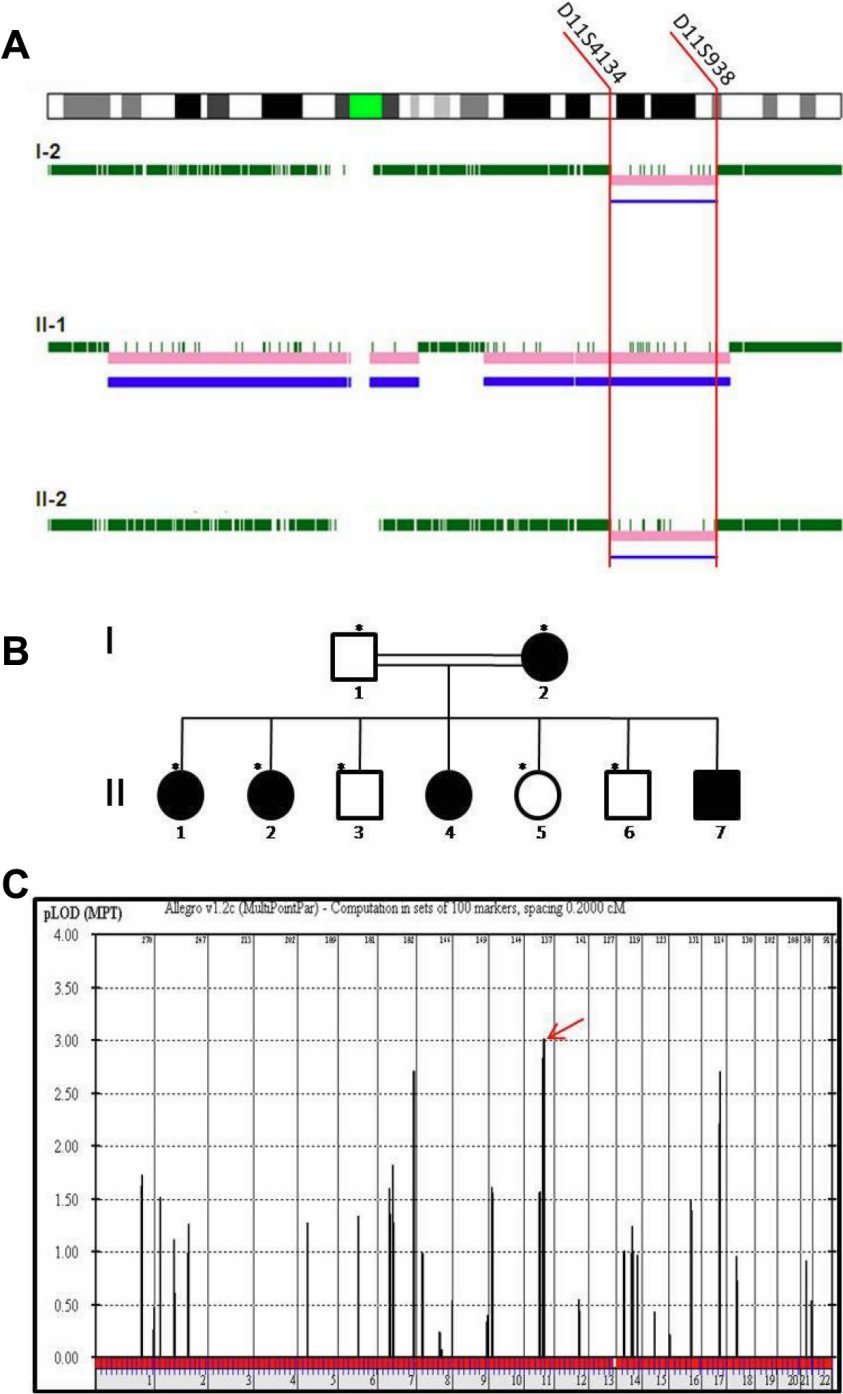
Autosomal recessive cataract family is linked to *CRYB*. **A**: CNAG analysis shows an area of homozygosity that is shared between affected family members on chromosome 11 as indicated by the pink bars. Microsatellite markers flanking the homozygosity region are shown. **B**: The two-generation pedigree shows the affected and unaffected members in shaded and open circles/boxes, respectively. Roman numbers denote generations, and Arabic numbers denote individuals within generations. **C**: Linkage analysis reveals the highest LOD score on chromosome 11 as the red arrow indicates.

A Saudi nuclear family with three affected individuals diagnosed with juvenile cataract was enrolled in this study (Figure 1). Informed consent was obtained from the participants in accordance with the study protocols approved by the ethics committees of the King Khaled Eye Specialist Hospital (Riyadh, Saudi Arabia) and King Faisal Specialist Hospital and Research Center (Riyadh, Saudi Arabia). The entire nuclear family received full ophthalmic evaluation.

### DNA Extraction

All family members gave approximately 5–10 ml of blood. DNA was extracted from whole blood using the Gentra reagent DNA Extraction Kit (Gentra, Valencia, CA) following the manufacturer’s instructions.

### Genotyping

DNA samples were processed following the instructions provided by the Affymetrix Gene Chip Human Mapping 250K Arrays (Affymetrix, Santa Clara, CA). In brief, 50 ng of high quality genomic DNA was digested with StyI followed by a ligation step with a universal adaptor. Ligated samples were then polymerase chain reaction (PCR)-amplified using primers complementary to the universal adaptor. PCR products were then fragmented and end-labeled with biotinlyated ddATP using terminal deoxynucleotidyl transferase followed by hybridization to the GeneChip Mapping 250K array (Affymetrix). Chips were processed with the Fluidic station and the GeneChip Scanner 3000 (Affymetrix). Average genotyping call rates for all samples used were approximately 92% (88%–95%).

### Homozygosity mapping and linkage analysis

Data generated by the 250K Affymetrix SNP (single nucleotide polymorphism) chip were used for linkage analysis and homozygosity mapping using Easylinkage (v5.08) software [10] and *Copy Number* Analyzer for Affymetrix GeneChip Mapping arrays (CNAG), respectively. CNAG uses SNP genotypes generated by a dynamic model (DM) algorithm for the detection of copy number changes and blocks of homozygosity. This tool allows multiple patient displays in a single window and facilitates rapid visualization of abnormalities, copy number variants, and common regions of homozygosity [11]. Easylinkage was used to confirm and calculate the maximum LOD score for the chromosomal regions identified by CNAG.

### Mutation analysis

The entire coding region and exon–intron boundaries of *CRYAB* were PCR-amplified. DNA sequence analysis was performed by dye termination sequencing (BigDye Terminator Cycle Sequencing v3.1 kit and the Prism 3730XL Genetic Analyzer; Applied Biosystems, Foster City, CA). DNA sequences were analyzed using the Seqman program of the DNASTAR analysis package (Lasergene, Madison, WI).

### Protein alignment

Protein sequence alignment was performed using the Multalin software v.5.4.1. Protein sequences from the following species were used to check for conservation at the mutated amino acid: *Homo sapiens*, *Pongo pygmaeus*, *Canis familiaris*, *Mus musculus*, *Rattus norvegicus*, *Equus caballus*, *Gallus gallus*, and *Danio rerio*.

## Results

### Clinical data

After informed consent, a nine-member nuclear family was studied. Three members (the mother, I-2, and two of her daughters, II-1 and II-2) had cataract surgery early in life. The parents, I-1 and I-2, were first cousins.

#### Patients with cataract history

Individual I-2, who was 35 years of age, had cataract surgery within the first six months of life for what was diagnosed as congenital cataract. The preoperative documentation of the type of cataract was unavailable to us. Ophthalmic examination was significant for bilateral aphakia and retinal dystrophic changes. Electroretinography confirmed depressed retinal function (both scoptopic and photopic).

The first child (II-1), 16 years of age, had bilateral cataract surgery for visually significant cataracts noted at 16 months of age and described as dense complete white cataracts. Ophthalmic examination revealed significant abnormalities in the right eye including aphakia and total retinal detachment while the left eye revealed nothing remarkable.

The second child (II-2), 14 years of age, had bilateral cataract surgery for visually significant nuclear cataracts at six years of age. Ophthalmic examination revealed bilateral pseudophakia and an unremarkable retinal examination.

#### Other family members

None of the other family members had ophthalmic complaints, and all had age-appropriate visual acuity. Ophthalmic examinations of the father (I-1) and the two sons, II-3 and II-6, were unremarkable. The third son (II-7) was a one-year-old and asymptomatic. He had an ophthalmic examination that was appropriate for his age. There was no evidence of significant lens opacity or other ocular abnormality. However, by retinoscopy, clinically insignificant fine opacities in the red reflect could be appreciated. Ophthalmic examination of the daughter, II-4 who was unaffected by history revealed visually insignificant lens (nuclear and cortical) opacities evident on careful slit-lamp examination. The remainder of her ophthalmic examination was unremarkable.

### Homozygosity mapping and linkage analysis

CNAG analysis of all affected members of the family showed a common region of homozygous SNPs on chromosome 11 (Figure 1A). Closer examination of the region using the UCSC genome browser showed that this region contains a previously reported cataract gene, *CRYAB*. This was also confirmed on linkage analysis, which revealed a LOD score of 3.0 for a locus on chromosome 11q21–23 including *CRYAB* (Figure 1C).

### Mutation analysis

**Figure 2:**
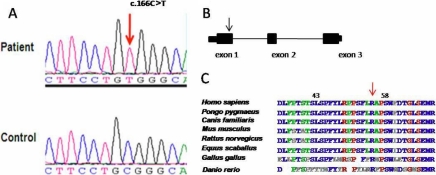
Novel missense mutation is identified in *CRYAB*. **A**: The sequence chromatogram shows the missense mutation, c.166C <T. **B**: The schematic diagram displays the genomic structure of *CRYAB*, and the position of the mutation identified in exon 1 is indicated by the black arrow. **C**: The resulting substitution occurs in a highly conserved amino acid residue, R56W, as shown in this cross-species alignment of amino acids.

Direct sequencing of *CRYAB* identified a c.166C <T transition resulting in a change in the amino acid from arginine to tryptophan (R56W; Figure 2A). This mutation was homozygous in the affected mother (I-2) and four of her children (II-1, II-2, II-4, and II-7). The unaffected father (I-1) was heterozygous for the mutation, which explains the pseudo-dominant inheritance pattern observed in this family. This sequence alteration was not found in 150 normal Saudi controls (300 chromosomes). In addition, R56 was found to be highly conserved across different species (Figure 2C).

## Discussion

We have identified a novel homozygous missense mutation in a family with an autosomal recessive form of congenital cataract. The recessive nature of the mutation is evident from the clinical assessment, which clearly indicated that only homozygous individuals were affected. In line with previous work showing retinal expression of *CRYAB* [12], the oldest affected individuals had retinal involvement. Therefore, several intriguing observations made by the current study are noteworthy. The first observation pertains to the recessive mode of inheritance of the mutation, which will have to be addressed in the context of the nature of this mutation and how it compares to those previously reported. αB-Crystallin is a small heat shock protein (sHSP) that has both structural (through its interaction with αA-crystallin in the lens fiber cells) and functional roles (as a chaperone that stabilizes and prevents aggregation of β- and γ-crystallins) [13]. Detailed analysis of its structure revealed the presence of NH_2_- and COOH-termini as well as an α-core crystallin domain. Intriguingly, the NH_2_-terminus, which lacks chaperone activity, has recently been shown to interact with Ksp, a kidney-specific cadherin, potentially playing a role in maintaining kidney tissue integrity [14]. Whether this binding capacity of the NH_2_-terminus extends to E-, P-, and N-cadherins that are known to be expressed in the lens is currently unknown. Ghosh and coworkers [13] have elegantly identified sequences that are important for subunit-subunit interactions and those involved in the chaperone activity of the protein. Interestingly, R56 lies almost in the middle of a short dual function sequence that serves both functions. Gain-of-function mutations where the mutant protein tends to self aggregate and dominant negative mutants were reported to result in both cataract as well as desmin-related myopathy [15]. Taken together, it is conceivable that the current mutation, which substitutes basic arginine for the non-polar tryptophan and thus reduces hydrophilicity, may only be sufficient in the homozygous form to disturb the interaction with αA-crystallin but may not be sufficient to disturb the chaperone activity. This would explain the recessive inheritance of the ophthalmic phenotype. However, this hypothesis may not be sufficient to explain the retinal involvement, which was not observed with any previously reported mutation. Here, we invoke a potential genotype-phenotype correlation as an explanation. It has been shown that *CRYAB* expression in the retina is increased in response to oxidative stress, and it has been postulated that this represents a protective mechanism against oxidative stress-induced apoptosis [16]. The latter proposition is based on the observation that αB-crystallin inhibits activation of caspase-3, a critical protease for apoptosis [17]. This result is supported by the finding that in *Cryab* knockout mice, there is increased susceptibility to apoptosis and retinal damage in response to infections [16]. Therefore, it is tempting to speculate that R56W impairs the inhibitory effect of αB-crystallin on caspase-3 and that the observed retinal phenotype may be the result of long-term impaired protection against apoptosis in response to oxidative stress. The fact that the retinal phenotype was only observed in the oldest patient (I-1) supports this hypothesis. However, we cannot exclude the possibility that the retinal phenotype in the mother (I-1) was due to another cause unrelated to *CRYAB*.

We note that in the family studied, there was a six-year-old girl (II-4) who was homozygous for the mutation but was clinically asymptomatic. However, slit-lamp examination revealed discrete visually insignificant lens opacities that were not present in other examined family members who were not homozygous for the mutation. The clinically insignificant fine opacities in the red reflex of II-7 were mostly likely lenticular. Thus, the homozygous mutation in this family may still be fully penetrant but with variable expressivity.

In summary, we report for the first time an autosomal recessive mutation in *CRYAB*, which results in cataract. We propose an allele-specific effect to explain the highly unusual associated retinal phenotype. Further studies are underway to experimentally test the effect of the mutation on αB-crystallin to enrich our understanding of the function and structure of this protein.
